# Early pathophysiology-driven airway pressure release ventilation versus low tidal volume ventilation strategy for patients with moderate-severe ARDS: study protocol for a randomized, multicenter, controlled trial

**DOI:** 10.1186/s12890-024-03065-y

**Published:** 2024-05-23

**Authors:** Yongfang Zhou, Jiangli Cheng, Shuo Zhu, Meiling Dong, Yinxia Lv, Xiaorong Jing, Yan Kang

**Affiliations:** 1 Department of Respiratory Care, West China Hospital of Sichuan University, Guoxue Alley 37#, Wuhou District, Chengdu, Sichuan 610041 China; 2 Department of Critical Care Medicine, West China Hospital of Sichuan University, Guoxue Alley 37#, Wuhou District, Chengdu, Sichuan 610041 China

**Keywords:** Acute respiratory syndrome distress, Airway pressure release ventilation, Low tidal volume, Mechanical ventilation, Randomized controlled trial

## Abstract

**Background:**

Conventional Mechanical ventilation modes used for individuals suffering from acute respiratory distress syndrome have the potential to exacerbate lung injury through regional alveolar overinflation and/or repetitive alveolar collapse with shearing, known as atelectrauma. Animal studies have demonstrated that airway pressure release ventilation (APRV) offers distinct advantages over conventional mechanical ventilation modes. However, the methodologies for implementing APRV vary widely, and the findings from clinical studies remain controversial. This study (APRVplus trial), aims to assess the impact of an early pathophysiology-driven APRV ventilation approach compared to a low tidal volume ventilation (LTV) strategy on the prognosis of patients with moderate to severe ARDS.

**Methods:**

The APRVplus trial is a prospective, multicenter, randomized clinical trial, building upon our prior single-center study, to enroll 840 patients from at least 35 hospitals in China. This investigation plans to compare the early pathophysiology-driven APRV ventilation approach with the control intervention of LTV lung-protective ventilation.

The primary outcome measure will be all-cause mortality at 28 days after randomization in the intensive care units (ICU). Secondary outcome measures will include assessments of oxygenation, and physiology parameters at baseline, as well as on days 1, 2, and 3. Additionally, clinical outcomes such as ventilator-free days at 28 days, duration of ICU and hospital stay, ICU and hospital mortality, and the occurrence of adverse events will be evaluated.

**Trial ethics and dissemination:**

The research project has obtained approval from the Ethics Committee of West China Hospital of Sichuan University (2019-337). Informed consent is required. The results will be submitted for publication in a peer-reviewed journal and presented at one or more scientific conferences.

**Trial registration:**

The study was registered at Clinical Trials.gov (NCT03549910) on June 8, 2018.

**Supplementary Information:**

The online version contains supplementary material available at 10.1186/s12890-024-03065-y.

## Introduction

Mechanical ventilation is a crucial component of the treatment of acute respiratory distress syndrome (ARDS) [1]. However, it has the potential to exacerbate lung injury through regional alveolar overinflation and/or repetitive alveolar collapse with shearing (atelectrauma) [2, 3]. In the conventional lung-protective ventilation strategy, achieving a balance between recruitment and over-distension is challenging due to the diverse lesion areas that require different pressures for reopening and have varying critical closing pressures for individual patients [4, 5]. Despite the development of numerous mechanical ventilation strategies over the past two decades, mortality rates for patients with moderate to severe ARDS remain unacceptably high, reaching 40–50% [6, 7].

Most mechanical ventilation strategies aimed at mitigating ventilator-induced lung injury (VILI) operate under the assumption that alveoli behave elastically, reaching an elastic load limit based on elastin/collagen interaction [8]. However, in reality, alveoli function as a viscoelastic system, exhibiting a lag between the onset or removal of stress and the initiation of volume change [9, 10]. From an anatomical standpoint, it is crucial to consider the presence of collateral respiratory pathways, such as the pores of Kohn, interalveolar septal pores, and canals of Lambert, which provide additional connections between adjacent alveoli, facilitating the redistribution of alveolar volume and pressure throughout the lung over time [11, 12]. This process promotes uniform ventilation and gas exchange within the lungs. Previous studies have indicated that alveolar recruitment and collapse not only rely on the magnitude of pressure applied to the lung but also on the duration for which the pressure is applied [13–15].

Considering the viscoelastic nature of the lung in ARDS, airway pressure release ventilation (APRV) was initially described in 1987 by Stock and colleagues as a groundbreaking form of mechanical ventilation. Instead of delivering tidal volume by elevating airway pressure above the positive end-expiratory pressure (PEEP), APRV entails the provision of continuous positive airway pressure with a brief intermittent release phase, enabling the release of only a partial lung volume and allowing spontaneous breathing throughout the high-pressure phase [16]. Theoretically, in the context of heterogeneous lung injury under APRV ventilation, the appropriate elevated baseline airway pressure (Phigh) at the safe target and prolonged duration of Phigh could optimally facilitate gradual alveolar recruitment over time, while averting overinflation. Moreover, the brief release phase (Tlow) allows for only a partial loss of lung volume to eliminate carbon dioxide, prevent alveolar collapse, and foster alveolar stability and homogeneity. Consequently, APRV emerges as an exemplary protective mechanical ventilation strategy for enhancing pulmonary function and mitigating lung injury [12, 17].

Animal experiments have illustrated that employing a physiology-driven approach to APRV, as opposed to low tidal volume ventilation (LTV), in ARDS animal models, enhances alveolar recruitment and gas exchange, mitigates lung injury, preserves surfactant protein and lung architecture, improves homogeneity without increasing lung stress and strain, and allows for spontaneous breathing [18–23]. This approach also decreases intrathoracic pressure, enhances systemic venous return, and mitigates the cardiovascular depressant effects of positive pressure [24–26]. Despite offering compelling physiological advantages over other ventilation modes, the clinical impact of APRV remains uncertain due to inconsistent outcome data, substantial heterogeneity in its application, and a dearth of convincing evidence [27].

In a single-center, randomized controlled trial, early physiology-driven APRV was compared with LTV in ARDS patients with a partial pressure of arterial oxygen and a fraction of inspired oxygen (PaO_2_/FiO_2_) ratio of ≤ 250mmHg [28]. The findings demonstrated that APRV significantly improved oxygenation and respiratory system compliance, reduced plateau airway pressure, decreased sedation requirement, and also led to hemodynamic improvement. A secondary analysis of existing data revealed that patients with moderate to severe ARDS derived greater benefit from mechanical ventilation with APRV than those with mild ARDS. To further investigate the early use of physiology-driven APRV in ARDS patients, our group has designed a multicenter randomized clinical trial to assess the impact of mechanical ventilation with APRV in patients with moderate-to-severe ARDS. This paper outlines the study procedures and planned analyses for this clinical trial, which is registered on ClinicalTrials.gov under number NCT03549910.

## Methods and analysis

### Trial conduct

We will procure informed consent from the legally authorized representatives. The trial has been approved by the Ethics Committee of West China Hospital of Sichuan University under approval number 2019 − 337, as well as the regional ethics committee of each participating site.

### Trial design

#### Study design

**Fig. 1:**
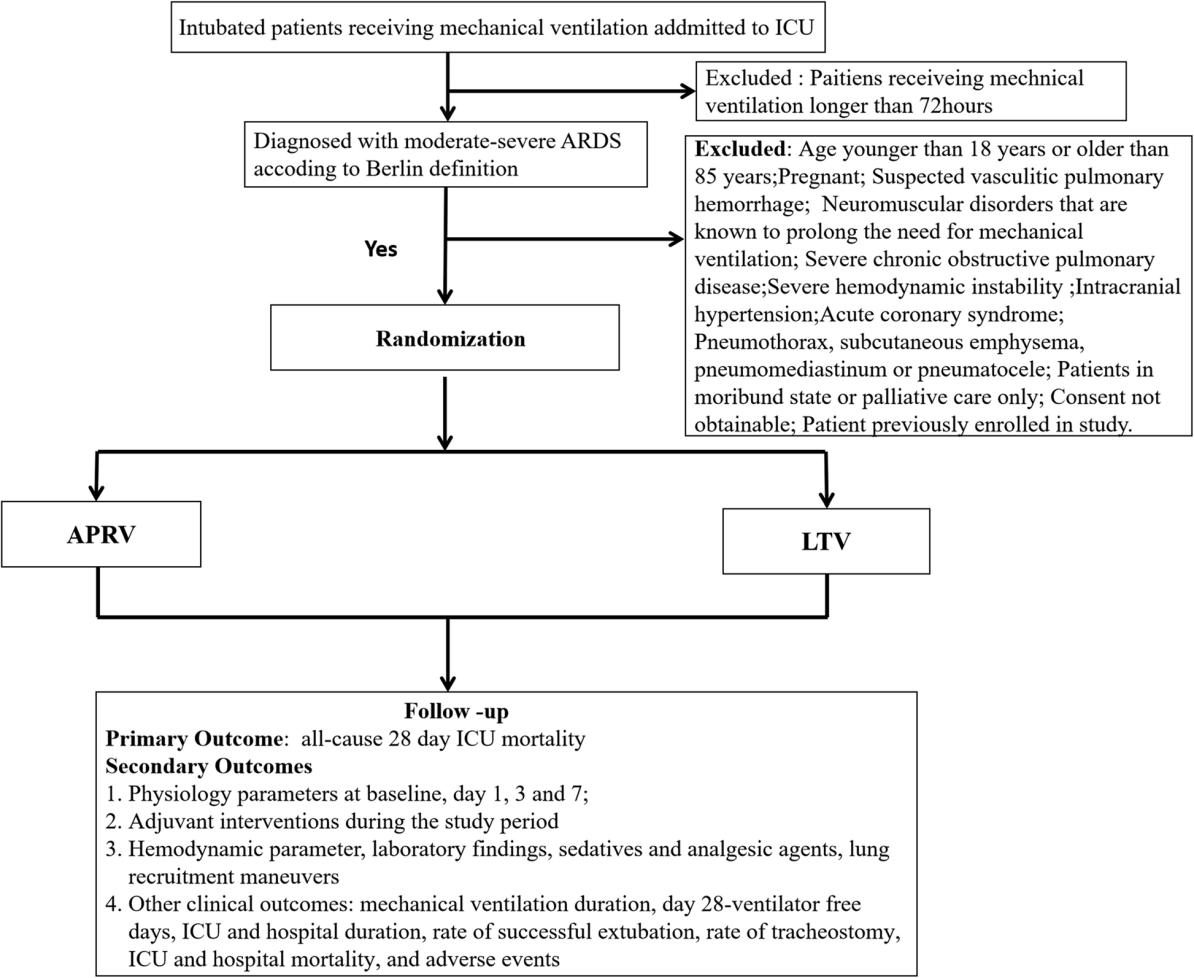
Study flow diagram

To test the hypothesis that early physiology-driven APRV ventilation will result in an improvement in the primary outcome of mortality until 28-day for patients with moderate to severe ARDS, we have developed a multicenter, prospective, randomized clinical trial (APRVplus trial). The overall study flow is depicted in Fig. 1.

#### Study population

The study will enroll adult patients aged 18 years and older who have been admitted to the intensive care unit (ICU) with moderate to severe ARDS, have undergone tracheal intubation, and received invasive mechanical ventilation for less than 72 h. The Berlin Conference definition will be utilized to identify patients with moderate-to-severe ARDS, including (A) hypoxemic respiratory failure with a PaO_2_/FiO_2_ ratio < 200 mmHg, (B) bilateral alveolar infiltrates on chest X-ray not present for more than 7 days, (C) respiratory failure not fully explained by cardiac failure or fluid overload, and (D) intubation on controlled ventilation and receiving PEEP ≥ 5 cmH_2_O [29].

Patients will be excluded from the trial if they meet any of the following criteria: (a) age over 85 years, (b) pregnancy, (c) suspected vasculitic pulmonary hemorrhage, (d) neuromuscular disorders known to prolong the need for mechanical ventilation, (e) severe chronic obstructive pulmonary disease, (f) severe hemodynamic instability (mean arterial pressure less than 60 mmHg despite sufficient fluid administration and use of high-dose vasoactive drugs (equivalent vasoactive drug dose: norepinephrine > 1.0 µg/kg/min)), (g) intracranial hypertension, (h) acute coronary syndrome, (i) pneumothorax, subcutaneous emphysema, pneumomediastinum, or pneumatocele, (j) patients in a moribund state or receiving palliative care only, (k) consent not obtainable, or (l) patients previously enrolled in another study.

Patients will be recruited from roughly 35 clinical sites in Mainland China with expertise in the identification and treatment of ARDS. Research investigators will provide training to the sub-research group at each sub-center on research protocols and implementation procedures. Coordinators at each participating site will assess potential candidates for enrollment. Once a patient is eligible for the study, a research investigator will obtain informed consent from the surrogate decision-maker. Subsequently, eligible patients will be enrolled and randomized, and initial ventilator adjustments will be made within 6 h.

#### Randomization and blinding

After enrollment, patients will be randomized in a 1:1 ratio to either the APRVplus group or the LTV group using a block randomization scheme. The random allocation list was crafted by a statistician uninvolved in the clinical aspects of the trial, utilizing a computer-generated random number list. Randomization will be stratified by enrollment site, pulmonary ARDS, and extrapulmonary ARDS. Assignment schedules were generated for each participating site, and allocation concealment was assured through a central web-based system (APRVplus.com) managed by Sichuan Zhikang Tech Co., Ltd. The treatment allocation for each patient will only be disclosed after their enrollment in the study.

As the intervention will be administered to critically ill patients on mechanical ventilation, most of whom are sedated, patient blinding is deemed unnecessary. Given that this is a nonpharmacological intervention, blinding the medical team is not feasible. Since there is no requirement for a committee to validate the study endpoint (death), outcome evaluators will not be blinded. However, the statisticians responsible for the analyses will be kept blinded to treatment groups for the duration of the study.

### Intervention

#### Ventilation strategy prior to randomization

In both groups, the goals of mechanical ventilation were to maintain PaO_2_ between 55 and 100 mmHg or pulse oximeter between 92% and 98%, PaCO_2_ between 30 and 50 mmHg, an arterial pH between 7.30 and 7.45, and limit plateau pressures no more than 30 cm of water (cmH_2_O). All patients initially received volume-assisted control ventilation (A/C-VCV) in accordance with the LTV ventilation strategy outlined by ARDSnet [30], prior to randomization into the APRV group or LTV group. Initially, tidal volume (VT) was set at 6mL/kg of predicted body weight (PBW), and PEEP was determined using the FiO_2_/PEEP table. Subsequently, the optimal PEEP was further adjusted based on oxygenation or compliance, at the discretion of the clinician. PEEP was incrementally increased by 2 cmH_2_O every 10 min, with measurements of PaO_2_ or respiratory system compliance taken at each step. The optimal PEEP was identified as 2 cmH_2_O below the level at which either PaO_2_ or compliance decreased by more than 10%. Alternatively, PEEP levels were adjusted using the pressure-volume (P-V) tool, with the optimal PEEP typically defined as 2 cmH_2_O above the lower inflection point (LIP) [31]. Tidal volume adjustments were made as follows: if the plateau pressure (Pplat) exceeded 30 cmH_2_O at a VT of 6 ml/kg predicted body weight and arterial pH was greater than 7.2, VT was reduced to 4–6 ml/kg predicted body weight. If Pplat was lower than 25 cmH_2_O, and patients experienced frequent stacked breaths or severe dyspnea, VT was adjusted to 6–8 ml/kg. Respiratory mechanics, including plateau pressure (Pplat), airway resistance (Rrs), and static compliance (Cstat) during a 0.5-second inspiratory pause, were monitored at least twice daily and after any changes in PEEP or VT.

#### Ensure sufficient cardiovascular system blood volume before randomization

Before randomization, effective cardiovascular system circulating blood volume should be evaluated. If the blood volume is insufficient, volume expansion treatment should be given to avoid hypovolemia. For shock patients, rapid fluid resuscitation and, if necessary, combined with vasoactive drugs will be used to achieve MAP ≥ 60 mmHg as soon as possible.

#### APRV group

In the APRVplus group, during the transition from the original ventilation with VCV mode to APRV, the following initial settings were applied: the high airway pressure (Phigh) was set at the Pplat measured at the previous VCV settings, not exceeding 30cmH_2_O; the low airway pressure (Plow) was set at 5cmH_2_O, representing the minimal pressure level similar to physiological transpulmonary pressure, and used to prevent atelectasis in accordance with established practice. The target tidal volume was set at 6-8 ml/kg PBW, and the duration of the release phase (Tlow) was adjusted as follows: Step one: The initial setting was 1.0 ~ 1.5 τ (expiratory time constant), which equals the product of the static compliance of the respiratory system and airway resistance (measured in lung volume unit L); Step two: Tlow was adjusted according to the expiratory flow-time curve, ensuring that the end-expiratory flow rate was ≥ 75% of the peak expiratory flow rate (PEFR); Step three: If the tidal volume was < 6 ml/kg PBW and patient-ventilator asynchrony occurred, Tlow was gradually extended, ensuring that the minimum end- expiratory flow rate was at least > 50% of PEFR; the release frequency was set at 10–14 frequency/min, or referenced to the original respiratory rate; the duration of Phigh (Thigh) was indirectly calculated based on Tlow and the release frequency. Initially, the spontaneous respiratory level was targeted as spontaneous minute ventilation (MVspont), approximately 30% of the total minute ventilation (MVtotal): severe ARDS (the ratio of PaO_2_: FiO_2_ < 100): MVspont less than 20% MVtotal, absent of dyspnea; mild to moderate ARDS (the ratio of PaO_2_: FiO_2_ > 100): MVspont: 20 ~ 60% of MVtotal, RR ≤ 35 times/min, no signs of respiratory distress.

Throughout the period of APRV ventilation, the APRV settings were adjusted based on the expiratory flow-time curve, lung mechanics and ventilation parameters, spontaneous breathing ventilation level, analgesia and sedation depth, patient-ventilator interaction, and arterial blood gas results, ensuring effective CO_2_ removal (PaCO_2_ 30 ~ 50mmHg) and adequate oxygenation (PaO_2_ 60 ~ 100mmHg). (For details on the APRV settings for titration, see Appendix).

#### Control group

In the control group, the upper limit for Pplat is set at 30 cmH_2_O, and the driving pressure is constrained to a maximum of 14 cmH_2_O. The target VT is 6 mL/kg PBW, and PEEP is adjusted based on optimal oxygenation, compliance, or P-V tool, with the flexibility to adjust VT within the range of 4–8 mL/kg PBW to prevent alveolar overextension and minimize patient-ventilator asynchrony. Specifically, in patients with a VT of ≥ 6 ml/kg PBW, the driving pressure exceeding 14 cmH_2_O and the Pplat above 25 cmH_2_O, or a Pplat exceeding 30 cmH_2_O with arterial pH greater than 7.2, VT is adjusted within the range of 4-6 ml/kg. VT can be gradually reduced in small decrements (e.g., 0.5 ml/kg PBW) over 1 h. Throughout the process of reducing VT, individual patient responses indicating intolerance to lower VT, such as tachycardia, hypotension, tachypnea, patient–ventilator dyssynchrony (e.g., double triggering), inspiratory airway pressure below PEEP indicating excess work of breathing, respiratory acidosis, and hypoxemia despite high FiO_2_, should be closely monitored over several hours. If signs of intolerance develop, adjunctive therapies, such as deep sedation, neuromuscular blockade or ECCO_2_R, should be considered to facilitate tolerance of low VT.

In cases of severe respiratory acidosis (pH<7.15), the respiratory rate was increased to 35 breaths per minute, with adjustments made in VT (Pplat target of 30 cmH_2_O may be exceeded), following the ARDSnet protocol. If severe respiratory acidosis persisted (pH<7.15), NaHCO_3_ could be administered.

If the PaO_2_:FiO_2_ ratio is less than 200 with FiO_2_ ≥ 0.5, the optimal PEEP should be checked using the above methods. If oxygenation does not improve, clinicians should assess and treat non-pulmonary causes of hypoxia, ruling out reversible causes and ensuring fluid optimization. In the presence of hypotension (mean arterial pressure less than 60mmHg) and pneumothorax occurrence with or without chest tube drainage, the clinician may modify PEEP levels according to the individual patient’s needs.

#### Recruitment maneuver

If the above interventions fail and patients without focal ARDS have been ventilated for less than 7 days, a stepwise recruitment maneuver can be performed. PEEP is increased in 5 cmH_2_O increments, allowing 30 s per step, until peak inspiratory pressure reaches 40 cmH_2_O, and then 40 cmH_2_O of pressure amplitude is applied for 40 s. In the descending period, PEEP is decreased by steps of 2 cmH_2_O every 5 min, and PaO_2_ or respiratory system compliance is measured at each step. The optimal PEEP is defined as 2 cmH_2_O above the level of PEEP where PaO_2_ or respiratory system compliance drops by more than 10% [32]. After the PEEP is set at the optimal level, a second recruitment maneuver is applied. If the PaO_2_:FiO_2_ ratio or respiratory system compliance increases by less than 10%, the recruitment maneuver should be stopped, and adjuvant therapies should be considered. For recruitment maneuver during APRV ventilation, Phigh and Plow were simultaneously increased in 5 cmH_2_O increments (allowing 30 s/step) until Phigh at 40 cmH_2_O, and then 40 cmH_2_O of pressure amplitude was applied for 40 s. In the descending period, Phigh and Plow were decreased by steps of 2 cmH_2_O/4 min and PaO_2_ was measured at each step. The optimal Phigh and Plow was defined as 2 cmH_2_O above the level of pressure, where PaO_2_ dropped more than 10%. After the optimal P high and Plow were set at the optimal level, a second recruitment maneuver was applied. If the PaO_2_:FiO_2_ ratio or respiratory system compliance increases by less than 10%, the recruitment maneuver should be stopped, and adjuvant therapies should be considered.

#### Co-interventions

Both intervention groups will receive standard analgesic and sedative treatment to ensure patient comfort while achieving the desired level of analgesia and sedation. The analgesia target level will be determined by the Critical-care pain observational tool (CPOT) score of 0 ~ 2 [33]. The sedation goal will be targeted as the Richmond Agitation Sedation Scale (RASS) score of -2 to 0 [33]. In the control group, clinicians will titrate the ventilator settings and the administration of analgesic and sedative drugs to achieve the optimal patient-ventilator interaction and sedation goal. In the APRV group, clinicians will adjust APRV settings and the dosages of analgesic and sedative drugs to achieve the desired level of spontaneous minute ventilation, analgesia, and sedation. If patients still exhibit anxiety, agitation, or respiratory distress after the ventilator settings have been optimized, deeper sedation (RASS scale < -2) will be administered.

As per usual sedation procedures, nurses will continuously monitor the depth of sedation and adjust the dosages of analgesic and sedative drugs to maintain the target level of analgesia and sedation. RASS scores will be recorded every 4 h (or more frequently when indicated) by the nursing staff to ensure accurate titration of the sedative infusion.

#### Adjuvant interventions for persistent hypoxemia

In cases of persistent severe hypoxemia (with no response to the assigned protocol and a PaO_2_:FiO_2_ ratio of < 150 mmHg during invasive mechanical ventilation for at least 12h), clinicians may consider implementing adjuvant interventions for hypoxemia at their discretion, such as prone positioning, neuromuscular blockade, or inhalation of nitric oxide, in both groups [34].

- 1) Prone positioning: If there are no contraindications, prone positioning can be performed for more than 12 h per day after careful preparation, following the criteria outlined by Guerin C, et al. [35]. Exclusion criteria for prone positioning include unstable spine, uncontrolled intracranial pressure, open abdominal injury, multiple trauma with skeletal/cervical traction, pregnancy, intra-abdominal pressure ≥ 20 mmHg, and severe hemodynamic instability.
- 2) Neuromuscular blockade: a If Pplat exceeds 30 cmH_2_O, and spontaneous inspiratory efforts are clinically detected, neuromuscular blockade is indicated. b Despite prioritizing optimization of ventilator settings, if patients exhibit rigorous respiratory effort, dyspnea, or breath stacking, neuromuscular blockade may be employed to prevent lung injury and facilitate smooth adaptation between the patient and the ventilator.
- 3) Inhalation of nitric oxide: If patients do not respond to the above therapies and continue to experience severe hypoxemia with a PaO_2_/FiO_2_ ratio < 150 and an increase in pulmonary artery pressure, administration of 5–20 ppm iNO with concentration may be considered, with reassessment 48h later.

All adjuvant interventions are utilized at the discretion of the clinician. Once the treatment goal of PaO_2_/FiO_2_ > 150 mmHg with FiO_2_ < 0.6 is achieved, clinicians may discontinue these interventions. If patients do not respond to the adjuvant interventions, the clinician may consider initiating ECMO.

#### Other treatments

During the treatment of ARDS, clinical doctors should manage patients according to best clinical practice evidence, closely and dynamically evaluate and solve pulmonary and extrapulmonary factors that cause or exacerbate hypoxia, such as anti-infection, optimized fluid therapy, maintaining appropriate hemoglobin levels, and optimize balance of oxygen supply and demand, actively prevent and treat complications.

### Weaning from mechanical ventilation

#### Weaning protocol in the LTV Group

Commencing the day after enrollment, patients in the LTV group will undergo a daily spontaneous awakening trial (SAT) followed by a spontaneous breathing trial (SBT) throughout the mechanical ventilation period [36]. If patients are under deeper sedation (RASS score < -2), they will be evaluated every morning by physicians using a daily SAT safety screen. In the absence of contraindications, such as severe hypoxemia, myocardial ischemia, hypertensive crisis, status asthmaticus, sustained agitation with increased use of sedation drugs, or treatment with neuromuscular blockers, patients will undergo an SAT trial, and sedative and analgesic infusions will be stopped until the patients are awake [37]. Analgesics for active pain will be continued. The awakening criteria include the ability of patients to perform three simple tasks: opening their eyes, squeezing the hand and moving fingers, and expressing discomfort [38]. If patients experience sustained agitation, marked dyspnea, SPO_2_ < 88% for ≥ 5 min, or arrhythmias, it is considered SAT failure. In such cases, bedside nurses will restart analgesics and sedatives at half the previous dose and titrate the medications to achieve the target sedation range [37]. These patients will be reassessed the following morning.

For patients under light sedation (RASS score of -2 to 0), sedative and analgesic infusions will be discontinued, while analgesics for ongoing pain management will be maintained. Patients who pass the SAT or those with RASS scores − 2 to 0 who have interrupted sedation will immediately undergo the SBT protocol. Respiratory therapists will manage patients with the SBT safety screen, and those who pass the criteria will undergo a 30-minute SBT trial with pressure support ventilation of 5–8 cmH_2_O, PEEP of 5 cmH_2_O, and FiO_2_ of ≤ 40% [39]. If the SBT trial is successful, physicians and respiratory therapists will decide on extubation. If the SBT is unsuccessful, pre-weaning settings will be resumed, and the patients will be reassessed the following morning.

The SBT safety screen criteria include improved cause of respiratory failure, FiO_2_ ≤ 0.40, PEEP ≤ 8 cmH_2_O, PaO_2_ ≥ 60 mmHg, acceptable spontaneous breathing efforts, systolic BP ≥ 90 mmHg, no significant use of vasopressors, and no neuromuscular blocking agents [37]. Successful tolerance for the SBT trial for up to 30 min is determined by specific criteria, including SpO_2_ ≥ 90% and/or PaO_2_ ≥ 60 mmHg, spontaneous VT ≥ 4 ml/kg PBW, respiratory rate ≤ 35/min, pH ≥ 7.3, hemodynamic stability (heart rate and blood pressure changing less than 20% from the previous level, or absence of acute cardiac arrhythmia), and absence of respiratory distress, such as respiratory rate exceeding 120% of baseline, significant accessory muscle use, abdominal paradox, diaphoresis, or pronounced dyspnea [39].

#### Weaning protocol in the APRV Group

- 1) First Stage: Once the goal for oxygenation has been achieved, FiO_2_ is gradually reduced to ≤ 0.6, followed by a stepwise decrease in Phigh by 1–2 cmH_2_O to ≤ 26 cmH_2_O, unless the patient’s oxygenation deteriorates.
- 2) Second Stage: After the improvement in the cause of respiratory failure, if pH ≥ 7.3, PaO_2_ > 70mmHg, SaO_2_ > 92%, FiO_2_ ≤ 40%, PaO_2_/FiO_2_ ≥ 200, Phigh is gradually and simultaneously reduced by 1–2 cmH_2_O and the release rate by 2 frequency twice daily unless the patient’s cardiopulmonary function deteriorates. Patients under deeper sedation (<-2) will have gradual and simultaneous reductions in analgesia and sedation based on the spontaneous minute ventilation target level and sedation goals combination.
- 3) Third Stage: When patients achieve the criteria with Phigh ≤ 20 cmH_2_O and FiO_2_ ≤ 40%, they will undergo the daily SBT trial, similar to the weaning protocol in the LTV group.

### Tracheostomy

Patients will be considered for tracheostomy if they meet any of the following criteria: (a) The duration of mechanical ventilation exceeding 2 weeks; (b) Upper respiratory airway obstruction, such as laryngeal edema; (c) Impaired airway protection, such as swallowing disturbances, ineffective cough, and massive airway secretions [40].

### The summary of APRV and LTV protocols is presented in Table 1.

#### Data collection and confidentiality

An independent research assistant will initiate the collection of baseline information. Diagnostic data, clinical characteristics, physiological parameter examinations, laboratory findings, and details regarding the dosage and type of sedatives, muscle relaxants, opiates, and vasopressors, as well as adjuvant therapies and outcome measures will be collected. All personal information will be kept confidential for research purposes only. All study data will be collected anonymously and assigned an individual study number on all case report forms. These will be managed using a central web-based, password-protected, encrypted electronic case report form (eCRF) system. Paper versions of the eCRF will be used only in the event of system malfunction. Project data sets will be stored on the Project Accept website and robust password-protected access systems will be implemented to secure all local databases. Investigators will have direct access to their own site’s data sets and can request access to data from other sites. To ensure confidentiality, data dispersed to project team members will be stripped of any identifying participant information where the participants' identifying information will be substituted with an unrelated sequence of characters. Data entry will be guided and overseen by full-time scientific research personnel. Independent data management will oversee the trial during the trial period in accordance with the predefined Charter for the DMSC.

**Table 1:** Summary of APRV and LTV protocols

	**APRV**	**LTV**
**Goals of mechanical ventilation**	PaO_2_: 55 -100 mmHg or SpO_2_: 92%-98%, PaCO_2_: 30-50 mmHg, and pH 7.30-7.45, and limit Pplat less than 30 cmH_2_O	
**Ventilator settings before randomization**	All patients initially A/C-VCV by ARDSnet, Vt was set at 6mL/kg/PBW initially, and PEEP was determined using the FiO_2_/PEEP table. Subsequently, the optimal PEEP was further adjusted based on oxygenation, compliance or P-V tool. Vt was adjusted to achieve goal of PaCO_2_ and pH.	
**Hemodynamic function management before randomization.**	Ensure sufficient cardiovascular system blood volume. For shock patients, rapid fluid resuscitation and, if necessary, combine with vasoactive drugs will be used to achieve MAP≥60 mmHg as soon as possible.	
**Ventilator settings after randomization**	**Phigh** was initially set at the Pplat measured at the previous VCV settings, not exceeding 30cmH_2_O.Then, Phigh was titrated to achieve the target PaO_2_and PaCO_2_.	**Tidal volume** (VT)was targeted at 6 mL/kg PBW. VT was adjusted within the range of 4–8 mL/kg PBW to achieve the goal of PaCO_2_ and pH, and target Pplat less than 30 cmH_2_O and driving pressure less than 14 cmH_2_O.
	**Plow** was 5 cmH_2_O, similar to physiological transpulmonary pressure	**PEEP** was adjusted based on the optimal oxygenation, compliance, or P-V tool.
	**Tlow** was initially set at 1.0~1.5 τ; then Tlow was adjusted according to the expiratory flow-time curve, ensuring that the end expiratory flow rate was ≥75% of the PEFR; If the Vt was < 6 ml/kg/PBW and patient-ventilator asynchrony occurred, Tlow was gradually extended, ensuring that the minimum end expiratory flow rate was at least > 50% of PEFR	/
	**Release frequency** was initially set at 10–14 frequency/min, or referenced to the original respiratory rate. Then release frequency was adjusted to achieve the goal of PaCO_2_ and PH, and the target spontaneous breath level. Thigh was only indirectly calculated based on Tlow and the release frequency	**Respiratory rate** was adjusted to achieve goal of PaCO_2_, PH (max 35 breaths per minute).
**Recruitment maneuver**	If the above interventions fail and patients without focal ARDS have been ventilated for less than 7 days, lung recruitment can be performed using the incremental PEEP approach.	
**Co-interventions**		
**Analgesia and sedation**	Analgesia goal: CPOT score of 0~2, Sedation: RASS score of -2 to 0. Nurses will continuously monitor the depth of sedation and adjust the dosages of analgesic and sedative drugs to maintain the target level of analgesia and sedation.	
**Jointly management of ventilator settings, analgesia, and sedation**	Clinicians will adjust APRV settings and the dosages of analgesic and sedative drugs to achieve the desired level of spontaneous minute ventilation, analgesia, and sedation.	Clinicians will titrate the ventilator settings and the administration of analgesic and sedative drugs to achieve the optimal patient-ventilator interaction and sedation goal.
**Adjuvant interventions**	In cases of persistent severe hypoxemia, if with no response to the assigned protocol and with a PaO_2_:FiO_2_ratio of <150mmHg for at least 12 hours, all adjuvant interventions can be utilized at the discretion of the clinician.	
**Prone positioning**	If there are no contraindications, prone positioning can be performed for more than 12 hours per day after careful preparation.	
**Neuromuscular blockade**	Neuromuscular blockade is indicated as the occurence of either of the following condition: a. If Pplat exceeds 30cmH2O, and spontaneous inspiratory efforts are clinically detected, b. Despite prioritizing optimization of ventilator settings, if patients exhibit rigorous respiratory effort, dyspnea, or breath stacking.	
**Inhalation of nitric oxide**	If patients do not respond to the above therapies and continue to experience severe hypoxemia with a PaO_2_/FiO_2_ratio<150 and an increase in pulmonary artery pressure, administration of 5-20 ppm iNO with concentration may be considered, with reassessment 48 hours later.	

#### Outcome

The primary outcome measure is mortality until day 28. Secondary outcome measures include physiological parameters at baseline, day 1, 3, and 7, encompassing ventilator settings and monitoring parameters, pulmonary mechanics such as plateau airway pressure, respiratory system compliance (Crs), and gas exchange parameters such as PaO_2_ and PaCO_2_. Additional variables include hemodynamic parameters, urine output, fluid balance, laboratory findings, and details regarding the dosage and type of sedatives and analgesics, vasopressors, lung recruitment maneuvers, and adjuvant therapies such as prone positioning, nitric oxide therapy, paralysis, and continuous renal replacement therapy. Outcome measures will encompass mechanical ventilation duration, ventilator-free days at day 28, ICU and hospital length of stay, rate of successful extubation, rate of tracheostomy, ICU and hospital mortality. Furthermore, any adverse events occurring during the study period, including barotrauma, ventilator-associated pneumonia, and other complications related to mechanical ventilation, will be collected and analyzed.

## An overview of the schedule of enrolment, interventions, and assessments is presented in Table 2.

### Sample size estimation and statistical analysis

#### Sample size

Based on previous research indicating a 37% mortality rate at ICU 28 days among moderate-severe ARDS patients receiving low tidal volume ventilation strategy [41], we postulated a 10% reduction in ICU 28-day mortality following the implementation of the early pathophysiology-directed APRV ventilation strategy compared to low tidal volume lung protective ventilation. Considering a hazard ratio of 0.66, a 90% power, and a two-sided significance level of 0.05, it is estimated that 762 patients are required to detect this difference. Accounting for approximately 10% loss in follow-up, enrollment of 840 patients (420 in each group) is necessary. An interim analysis will be conducted once 50% of the sample size is reached.

**Table 2:**
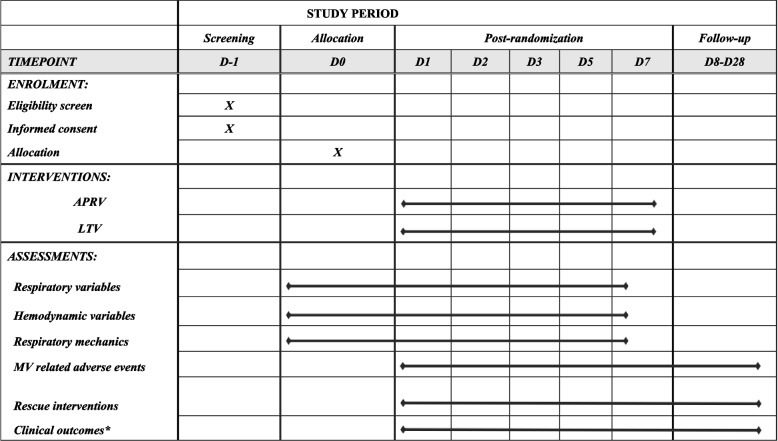
Overview of the schedule of enrolment, interventions and assessments

*APRV* airway pressure release ventilation, *LTV* low tital ventilation

#### Statistics

Continuous variables will be presented as mean and standard deviation, or median and interquartile ranges (IQR). Categorical data will be described with counts and percentages. Continuous variables with a normal distribution will be analyzed using the Student’s t-test, while those with a non-normal distribution will be compared using the Kruskal–Wallis analysis of variance. Dichotomous or nominal categorical variables will be analyzed using either the Pearson Chi-square or Fisher’s exact test. The trend over time in oxygenation and respiratory mechanics will be compared between the LTV group and APRV group using repeated-measures analysis. A two-sided *P* value of < 0.05 is considered statistically significant.

All analyses will adhere to the intention-to-treat principle (per protocol analysis). The primary outcome will be assessed using Kaplan-Meier curves and the ratio will be calculated with a 95% confidence interval using the Cox proportional hazard model. We plan to conduct a sensitivity analysis for the primary outcome using multiple imputation techniques only if follow-up data of 1% or more of the patients is lost. Interim analyses will be performed after recruiting half of the planned sample size to evaluate effects on clinical outcomes. The data monitoring committee would consider stopping the trial if there was evidence of harm with a one-sided *P* value < 0.01.

We will estimate the effects of the intervention using generalized linear models employing gamma distributions for lengths of ICU and hospital stay) or a truncated Poisson distribution (ventilator-free days). The treatment effect on 28-day mortality will be in subgroups based on PaO_2_:FIO_2_ (≤ 100 vs. > 100 mmHg) and pulmonary vs. extrapulmonary ARDS.

All analyses will be performed using the R software and subgroups will be evaluated using the chi-square test for homogeneity, we will employ the statistical software R Project Version 4.1.3 for Windows to carry out the analyses.

#### Data monitoring committee

A Data Monitoring Committee comprising independent epidemiologists and intensivists will be established to oversee the study. The committee will be responsible for assessing the potential risks and benefits associated with the research, and will provide recommendations on whether to continue with the planned study or cease recruitment. This decision will be based on evidence indicating a higher mortality rate in the experimental group compared to the control group. Any safety-related adverse events will be promptly reported to the site’s Institutional Review Board (IRB). Site investigators will be informed of the event and will subsequently submit a comprehensive written report to the local IRB. Sites will notify the research team of any related adverse events within a week of their discovery and complete the appropriate eCRF. The research team will provide the Data Monitoring Committee with summaries of all reports at least biannually. The Committee will convene via teleconference or in-person meetings at 25%, 50%, and 75% of enrollment, or earlier if necessary, to review adverse events.

#### Ethics and dissemination

The protocol has received approval from the Ethics Committee of West China Hospital of Sichuan University (approval number 2019 − 337). Additionally, approvals for the protocol and informed consent documents are obtained from the Institutional Review Board of each participating institution before enrolling study participants. Written informed consent is required and obtained from legally authorized representatives at the respective study site. Trial methods and results will be reported according to the Consolidated Standards of Reporting Trials (CONSORT) 2010 guidelines [42]. The primary outcome of the study will be published as the first article and additional results extrapolated from the data could be published in separate articles. The findings of this study will be disseminated in peer-reviewed journals, presented at scientific conferences, and shared with the practice.

#### Protocol amendments

Any modifications to the study will prompt simultaneous protocol adjustments, which will be promptly submitted for approval to the institutional review board. The changes will only be executed following the endorsement of the ethical committee. Upon approval, the amendments will be disseminated to other participating sites, and ClinicalTrials.gov will be promptly updated regarding any significant changes. If required, the study team will provide protocol training for the amendments.

## Discussion

Despite the potential benefits of the physiologically sound approach demonstrated by experimental studies, it is not commonly utilized in clinical practice for patients with ARDS due to a lack of evidence and gaps in the literature. Data is limited to several small, single-center clinical trials where APRV was inconsistently applied, and some trials faced challenges with protocol adherence and were prematurely halted, resulting in controversial results [43–46]. To our knowledge, this study is the first multicenter, large sample, randomized clinical trial to investigate the potential benefits of APRV on clinically relevant outcomes in patients with moderate-to-severe ARDS. We hypothesize that the early, physiology-guided approach to utilizing APRV will result in improved 28-day mortality rates compared to the standard of care, which delivers LTV lung-protective ventilation. Additionally, this investigation will provide information on important physiological measurements, other clinical outcomes, adverse events associated with these two strategies, as well as the potential value of using relevant biomarkers and key physiological data as surrogate outcome markers for mortality in ARDS. Ultimately, the results of this study will provide essential information on the scientific merit of APRV. If the experimental intervention proves superior to standard care, this will provide an evidence base to support the use of APRV in clinical practice, which would have significant clinical relevance for mechanically ventilated patients with ARDS.

The primary strength of this trial lies in the development of a detailed ARDS pathophysiology-driven APRV protocol, which is based on our wealth of successful experience in applying APRV for ARDS patients over the past decade. Our previous single-center randomized trial has validated the safety and efficacy of this protocol [28]. The current trial builds upon this foundation by enrolling more patients and involving multiple centers, with a more serious primary endpoint than our previous study. This will allow us to demonstrate the feasibility of implementing our physiology-driven APRV protocol across multiple centers, increasing the generalizability of the study results to the adult mechanically ventilated ARDS population. These results may be useful in the development of guidelines in the future.

There are several significant limitations to consider in the planned investigation. A primary concern is the intricacy of the APRV protocol, which demands a higher level of knowledge and skill compared to other modes. Extensive efforts have been made to ensure that all participating centers possess a comprehensive understanding of the APRV protocol. Initially, we conducted a thorough survey among Chinese ICU clinicians to assess the status of each participating site, including their current utilization and comprehension of APRV, as well as their mechanical ventilation strategies in managing ARDS. This was essential for selecting appropriate sites for the trial and implementing tailored training programs for each site. The training regimen encompasses didactic lectures covering basic respiratory physiology associated with ARDS and APRV, as well as the APRV and LTV protocols. Additionally, hands-on experience and simulation training are provided to interpret the utilization of the APRV and LTV protocols, along with troubleshooting procedures. The research group has developed a comprehensive handbook of training materials, online training videos, and knowledge- and scenario-based tests. Investigators and their teams at all participating sites will undergo recurrent training until they achieve a passing score of ≥ 85.

In addition, training sessions will be conducted for investigators and their teams at the outset of the study, with subsequent sessions provided as needed at each site. Furthermore, respiratory therapists from our center will guide mechanical ventilation management to investigators and their teams via the online platform WeChat throughout the study, ensuring accurate utilization and compliance with protocols and standards of good clinical practice. Finally, monitors will conduct integrity and accuracy checks for data in the eCRF system to guarantee patient safety and appropriate data collection.

## Conclusion

We have developed a comprehensive protocol and launched the first multicenter randomized trial to evaluate the potential benefits of the APRVplus protocol on clinically relevant endpoints in patients with moderate to severe ARDS. This protocol represents significant advancements from our previous single-center study through the increased number of enrolled patients and participating centers, as well as a serious patient-centered primary outcome. In addition to providing crucial insights into the safety and efficacy of APRV utilization in patients with moderate-to-severe ARDS, the results of this clinical trial will establish the viability of APRV implementation in routine clinical practice. If a favorable effect is demonstrated, it could have a profound impact on the future of mechanical ventilation for patients with ARDS.

## Trial update

The initial patient was randomized on December 10, 2020, and recruitment is currently ongoing. We anticipate that the study will be completed by the end of December 2024.

### Supplementary Information

Supplementary Material 1.

## Data Availability

Any data collected during this study can be acquired from the corresponding author upon a reasonable request.

## Abbreviations

A/C-VCV: volume-assisted control ventilation
APRV: airway pressure release ventilation
ARDS: acute respiratory distress syndrome
CONSORT: the Consolidated Standards of Reporting Trials
CPOT: the Critical-care pain observational tool
Cstat: static compliance
ICU: intensive care units
LIP: lower inflection point
LTV: low tidal volume
MVspont: spontaneous minute ventilation
MVtotal: total minute ventilation
PaO_2_/FiO_2_: partial pressure of arterial oxygen and a fraction of inspired oxygen
PEEP: positive end-expiratory pressure
Phigh: the high airway pressure
Plow: the low airway pressure
Pplat: plateau pressure
P-V tool: pressure-volume tool
RASS: the Richmond Agitation Sedation Scale
RCT: randomized clinical trial
Rrs: airway resistance
SAT: daily spontaneous awakening trial
SBT: spontaneous breathing trial
Thigh: the duration of Phigh
Tlow: the duration of Plow
VILI: ventilator-induced lung injury
VT: tidal volume
