# Supplementary material for: Early pathophysiology-driven airway pressure release ventilation versus low tidal volume ventilation strategy for patients with moderate-severe ARDS: study protocol for a randomized, multicenter, controlled trial
Source: BMC Pulm Med. 2024 May 23;24:252. doi: 10.1186/s12890-024-03065-y (PMC11112826; doi:10.1186/s12890-024-03065-y)
Supplement: Supplementary file 1 — Supplementary Material 1. [file 12890_2024_3065_MOESM1_ESM.docx]

**Appendix：APRV protocol**

Patients allocated in airway pressure release ventilation (APRV) group were firstly titrated the optimal positive end-expiratory pressure (PEEP) using P-V tool or the best respiratory complicance method and tidal volume under volume-assisted control ventilation (A/C-VCV) ventilation as described in low tidal volume (LTV) group. The plateau pressure (Pplat), respiratory static compliance (Cstat), and airway resistance (Rrs) were measured under the optimal PEEP and the tidal volume (VT) of 6ml/kg PBW. Then, the patient was transitioned to APRV. Throughout the period of APRV ventilation, the APRV settings were adjusted based on the expiratory flow-time curve, lung mechanics and ventilation parameters, spontaneous breathing ventilation level, analgesia and sedation depth, patient-ventilator interaction, and arterial blood gas results, ensuring effective CO_2_ removal (PaCO_2_ 30~50mmHg) and adequate oxygenation (PaO_2_ 60~100mmHg).

1. **APRV initiation settings**

**Table 1. Initiation settings of APRV**

Initiation settings of APRV

1. Under original ventilation with VCV mode, titrate the optimal PEEP as described in LTV group with VT of 6ml/kg PBW, and then measure Pplat, Rrs and Cstat parameters;
2. Phigh：if Pplat less than 30 cm H_2_O, Phigh= Pplat; if Pplat exceeds 30 cm H_2_O, Phigh= 30 cm H_2_O;
3. Plow：5cm H_2_O (Use a minimum PEEP level to prevent atelectasis as our usual care);
4. Tlow: (the goal of VT: 6-8ml/kg PBW)：
5. First step：Calculation time constants(τ), τ=R(cmH_2_O/L/S)×C( L/cmH_2_O)；Initial Tlow: 1.0τ-1.5τ ;
6. Second step: Titration of Tlow to more than or equal to 75% PEFR (Peak expiratory flow rate);
7. Third step: If Vt is less than 6 ml/kg and the patient–ventilator asynchrony occurred, it is allowed to gradually extend Tlow to maintain a termination of expiratory flow rate more than 50% PEFR.
8. Release frequency: set initial release frequency according to the following table

| Original respiratory rate /min | 10-14 | 15-18 | 19-22 | 23-24 | 25-26 | 27-28 | 29-30 | 31-35 |
| --- | --- | --- | --- | --- | --- | --- | --- | --- |
| Initial release frequency/min | 10 | 11-12 | 13-14 | 15-16 | 17-18 | 19-20 | 21-22 | 23-25 |

1. Trigger: Flow triggering at 2-3L/min, if there is a false triggering, change to pressure triggering at 1-3cmH_2_O.
2. FiO_2_: Same as prior mode;
3. Preserve partial spontaneous respiratory, target spontaneous respiratory level as spontaneous minute ventilation (SV) to total minute ventilation (MVtotal):

a. Mild to moderate ARDS (PaO_2_/FiO_2_ >100): SV equal to 20-60% MVtotal, and RR ≤ 35 per min, absent of dyspnea；

b. Severe ARDS (PaO_2_/FiO_2_ ≤100)：SV < 20% MVtotal, and RR ≤ 35 per min, absent of dyspnea.

Note: VCV volume assisted-control ventilation, Pplat plateau airway pressure, Rrs respiratory system resistance, Cstat static respiratory system compliance, Phigh the high airway pressure, Plow the low airway pressures, PEEP positive end-expiratory pressure, VT tidal volume, SV spontaneous minute ventilation, ARDS acute respiratory distress syndrome, MVtotal total minute ventilation.

1. **SB maintenance and sedation titration during the APRV ventilation**

**Table 2-1. SB maintenance and sedation titration for light to moderate ARDS：**

**Target SV (20%~60%MVtotal) and Sedation (RASS -2~0)**

Sedation↓

SV<20% and Sedation <-2

SV<20% and Sedation ok

If pH≥7.3，F↓to the SV target

If pH＜7.3，Sedation↓，when necesary F↑(maximum 30 per minute)，and PaCO_2_≥30mmHg；

If pH persist＜7.3, consider other measures.

SV>60% and Sedation ok

If pH≤7. 45, F↑to the SV target

If pH>7.45,

sedation↑，when necesary F↑to the SV target.

SV>60% and sedation＜-2

Or RR≥35 breaths/min

If pH＞7.45，sedation↑，when necesary, F↑to the SV target, even considering paralysis.

If pH≤7.45, F↑to the SV target

**Table 2-2. SB maintenance and sedation titration for severe ARDS**

Phigh＞28cmH_2_O, target SV for 0.

Phigh≤28 cmH_2_O：

SV (10%~20%MVtotal) and Sedation (RASS -2~0)

In the presence of SB, apply paralysis

SV>20% and Sedation ok

If pH≤7.45, F↑

to the SV target

If pH>7.45,

sedation↑，when necesary F↑ to the goal of SV.

SV<10% and Sedation <-2

Sedation↓

SV>20% and sedation＜-2

Or RR≥35 breaths/min

If pH＞7.45, sedation↑，when necesary, F↑to the SV target, even considering paralysis.

If pH≤7. 45, F↑ to the SV target

If pH ≥7.3，F↓to

the SV target

If pH＜7.3, Sedation↓，when necesary F↑(maximum 30 per minute)，and PaCO_2_≥30mmHg；

If pH persist＜7.3, consider other measures.

SV<10% and Sedation ok

Note: APRV airway pressure release ventilation, SB spontaneous breathing, MVtotal total minute mechanical ventilation, SV spontaneous minute mechanical ventilation, RASS the Richmond Agitation Sedation Scale NMB Neuromuscular blokade.

1. **Titration of APRV settings**

**Table3. Titration of APRV settings**

**Hypercapnia**

1. Ensure patient is not over sedated and achieve the SV level.
2. Increase release minute ventilation：

➀ Increase release frequency:

Increase release frequency by 1-2 rate per increment (Max 30 frequencies/minute)

➁ Increase release volume

a. Increase T_low_ 0.05-0.1s per increment (at least >50% PEFR) and keep Vt ≤8ml/kg PBW

b. Increase △P (Phigh-Plow)

Increase Phigh by 1- 2cmH_2_O per increment (max30cmH_2_O);

Decrease Plow by 1-2cmH_2_O as necessary;

1. Severe hypercapnia (PH≤7.2, PaCO_2_>60mmHg)

Increase △P simultaneously increase release frequency

1. If hypercapnia is not responsive to all the above adjustments,

APRV was transmitted to other treatment.

1. Increase Phigh by 2cmH_2_O per increment (max 30cmH_2_O); When Phigh ≥ 28cmH_2_O, firstly increase FiO_2_ to maximum of 100%

(2) With CO_2_ retention, improve alveolar ventilation

➀ Increase release frequency by 1-2 rate per increment (max 30 per min)

➁ Increase T_low_ 0.05-0.1s per increment (at least >50% PEFR) and keep Vt ≤8ml/kg PBW

(3) Without CO_2_ retention, prolong Thigh:

➀ Decrease release frequency by 2 rate per decrement (one adjustment within 2 hours);

➁ Decrease T_low_ 0.05-0.1s (Vt > 4ml/kg PBW);

➂ Increase Plow by 2-4 cmH_2_O as necessary;

(4)Ensure the sedation and SV target level;

a. If PaO_2_ < 60 mmHg after above adjustments, increase FiO_2_;

b. If PaO2:FiO2 ratio <100mmHg for at least 12 hours after above treatment, combine prone potion;

1. If hypoxemia is not responsive to all the above adjustments, APRV was transmitted to other treatment.

**Hypoxemia**

**According to respiratory mechanics, ventilation parameters, arterial blood gas analysis, hemodynamic parameters.**

Note: APRV airway pressure release ventilation, SV spontaneous breath minute ventilation, PEFR peak expiratory flow rate, Phigh, Plow, Thigh, Tlow and FiO_2_ were titrated based on interpretation of the measured respiratory mechanics at least twice daily, such as expiratory flow waveform, respiratory system compliance, airway resistance, release volumes (also defined as monitoring tidal volumes), MVtotal, SV, PEEP, Ppeak, Pplat, and arterial blood gases, chest imaging and bedside lung ultrasound.

1. **Recruitment maneuver during APRV ventilation**

If the above interventions fail and patients without focal ARDS have been ventilated for less than 7 days, lung recruitment can be allowed. PEEP high (Phigh) and PEEP low (Plow) were simultaneously increased in 5 cmH_2_O increments (allowing 30 seconds/step) until P high at 40 cmH_2_O, and then 40cmH_2_O of pressure amplitude was applied for 40seconds. In the descending period, P high and Plow were decreased by steps of 2 cmH_2_O / 4 min and PaO_2_ was measured at each step. The optimal P high and Plow was defined as 2 cmH_2_O above the level of pressure, where PaO_2_ dropped more than 10%. After the optimal P high and Plow were set at the optimal level, a second recruitment maneuver was applied.

| **Table 4：Weaning** | | | | | | | | | | | | | | | | |
| --- | --- | --- | --- | --- | --- | --- | --- | --- | --- | --- | --- | --- | --- | --- | --- | --- |
|  | **First stage**  **Safe mechanical ventilation management** | | | | | | | | | | **Second stage**  **The feasibility of weaning management** | | | | | **Third stage**  **Spontaneous breathing trial** |
| Phigh cmH_2_O | **30** | **29** | **28** | **28** | **28** | **28** | **28** | **27** | **27** | **26** | **26** | **25** | **24** | **23** | **22~20** | **20~18**  **SBT** |
| FiO_2_ | **1.0** | **1.0** | **1.0** | **0.9** | **0.8** | **0.7** | **0.6** | **0.6** | **0.5** | **0.5** | **0.4** | **0.3** | **0.3** | **0.3** | **0.3** | **0.3** |
| Note | When the oxygenation target was achieved: If Phigh>28 cmH_2_O, firstly, it was stepwise decreased by 1cmH_2_O to 28 cmH_2_O; and then gradually reduce FiO_2_ to ≤0.7. Then Phigh and FiO_2_ were alternately and gradually reduced to 26cmH_2_O and 0.5 respecttively.  During the process of down-regulating the support level of mechanical ventilation, if the patient’s cardiopulmonary function deteriorated (SpO_2_ drops≥2% or arterial PaO_2_ drops≥5%, HR or MAP changes≥20%), the previous ventilator settings will be restored. | | | | | | | | | | After the cause of respiratory failure has been improved, and Phigh≤26 cmH_2_O，pH ≥7.3, PaO_2_>70mmHg, SaO_2_>92%, FiO2≤40%: Phigh is gradually and simultaneously reduced by 1-2 cmH_2_O and the release rate by 2 frequency twice daily, unless the patient's cardiopulmonary function deteriorates（SpO_2_ drops ≥2% or PaO_2_ drops ≥5%，HR or MAP changes ≥20), the ventilator settings will be restored.  Patients under deeper sedation (<-2) will undergo gradual reductions in the depth of analgesia and sedation based on the spontaneous minute ventilation target and sedation goal. | | | | | When patients achieve the criteria with Phig≤20 cmH_2_O  and FiO_2_≤40%, clinicians and/or respiratory therapists will perform daily SBT screening and assess whether patients meet the SBT screening criteria or extubation criteria according to the weaning guideline of AARC. If SBT fails, restore the ventilator settings and reevaluate the next day. |

1. **Weaning**
2. **Other treatments**

During the treatment of ARDS, clinical doctors should manage patients according to best clinical practice evidence, closely and dynamically evaluate and solve pulmonary and extrapulmonary factors that cause or exacerbate hypoxia, such as anti-infection, optimized fluid therapy, maintaining appropriate hemoglobin levels, and optimize balance of oxygen supply and demand, actively prevent and treat complications.

**Abbreviations**

A/C-VCV: volume-assisted control ventilation

APRV: airway pressure release ventilation

ARDS: acute respiratory distress syndrome

CPOT: the Critical-care pain observational tool

Cstat: static compliance

LTV: low tidal volume

MVspont: spontaneous minute ventilation

MVtotal: total minute ventilation

PaO2/FiO2: partial pressure of arterial oxygen and fraction of inspired oxygen

PEEP: positive end-expiratory pressure

Phigh: the high airway pressure

Plow: the low airway pressure

Pplat: plateau pressures

P-V tool: pressure-volume tool

RASS: the Richmond Agitation Sedation Scale

Rrs: airway resistance

SBT: spontaneous breathing trial

Thigh: the duration of Phigh

Tlow: the duration of Plow

VT: tidal volume
